# Histological variants of pancreatic ductal adenocarcinoma: a survival analysis

**DOI:** 10.1007/s00423-024-03506-6

**Published:** 2024-10-19

**Authors:** Axel Bengtsson, Roland Andersson, Daniel Ansari

**Affiliations:** 1 Department of Surgery, Clinical Sciences Lund, Lund University, Skåne University Hospital, Lund, SE-221 85 Sweden; 2 Department of Research and Development, Region Kronoberg, Växjö, Sweden

**Keywords:** Pancreatic ductal adenocarcinoma, Histological subtypes, Survival, SEER

## Abstract

**Purpose:**

Pancreatic ductal adenocarcinoma (PDAC) can be classified into distinct histological subtypes based on the WHO nomenclature. The aim of this study was to compare the prognosis of conventional PDAC (cPDAC) against the other histological variants at the population level.

**Methods:**

The Surveillance, Epidemiology and End Results (SEER) database was used to identify patients with microscopically confirmed PDAC. These patients were divided into 9 histological subgroups. Overall survival was assessed using the Kaplan-Meier method and Cox regression models stratified by tumor histology.

**Results:**

A total of 159,548 patients with PDAC were identified, of whom 95.9% had cPDAC, followed by colloid carcinoma (CC) (2.6%), adenosquamous carcinoma (ASqC) (0.8%), signet ring cell carcinoma (SRCC) (0.5%), undifferentiated carcinoma (UC) (0.1%), undifferentiated carcinoma with osteoclast-like giant cells (UCOGC) (0.1%), hepatoid carcinoma (HC) (0.01%), medullary carcinoma of the pancreas (MCP) (0.006%) and pancreatic undifferentiated carcinoma with rhabdoid phenotype (PUCR) (0.003%). Kaplan-Meier curves showed that PUCR had the worst prognosis (median survival: 2 months; 5-year survival: 0%), while MCP had the best prognosis (median survival: 41 months; 5-year survival: 33.3%). In a multivariable Cox model, several histological subtypes (i.e. CC, ASqC, SRCC, UCOGC) were identified as independent predictors of overall survival when compared to cPDAC.

**Conclusion:**

PDAC is a heterogenous disease and accurate identification of variant histology is important for risk stratification, as these variants may have different biological behavior.

## Introduction

Pancreatic cancer is the third leading cause of cancer-related death [1]. By 2040, the incidence is projected to have nearly doubled and pancreatic cancer is expected to become the second deadliest cancer [2]. The technical advancements in operative techniques and developments of new chemotherapy regimens have improved oncological outcomes, but resulted only in marginal improvements in overall survival [3, 4]. The variable response rates may be attributed to biological heterogeneity. Genomic analyses of bulk tumor tissue, as well as single cell RNA sequencing, have identified several distinct molecular subtypes of pancreatic cancer [5–8]. While these molecular insights may allow for more personalized treatment strategies, they have yet to be translated to the clinical setting.

In routine clinical practice, histological classification remains the gold standard for perioperative management. Most pancreatic cancer (> 90%) are histologically classified as pancreatic ductal adenocarcinoma (PDAC). According to the WHO classification [9], PDAC can be further subdivided into conventional pancreatic ductal adenocarcinoma (cPDAC), adenosquamous carcinoma (ASqC), colloid carcinoma (CC), hepatoid carcinoma (HC), medullary carcinoma of the pancreas (MCP), pancreatic undifferentiated carcinoma with rhabdoid features (PUCR), signet ring cell carcinoma (SRCC), undifferentiated carcinoma (UC) and UC with osteoclast-like giant cells (UCOGC).

Variant histology not only presents diagnostic challenges, but also carries potential implications concerning prognosis and treatment. The optimal management of histological variants remains uncertain due to limited underlying evidence. Published data are based on small retrospective series, or even case reports [10], and patients with variant histology are often excluded from clinical trials. Therefore, clinical management of histological variants may often rely on extrapolation of data from cPDAC.

The aim of this study was to compare the clinical features and prognosis of cPDAC against the other less common histological variants using a large, population-based registry.

## Materials and methods

### Data source

Source material for the study was the Surveillance, Epidemiology and End Results (SEER) program. SEER is a population-based cancer registry from the United States. Using SEER*Stat version 8.4.3 (November 2022 data submission), data from all cancer registries participating in the SEER program were retrieved. The study followed the STROBE guidelines [11].

### Study cohort

All patients with pancreatic cancer registered in the SEER database between 2004 and 2020 were identified on the basis of the International Classification of Diseases for Oncology, third edition (ICD-O-3) topography codes (C25.0 to C25.9), as well as histological codes: cPDAC (8140/3, 8500/3), ASqC (8560/3), CC (8480/3), HC (8576/3), MCP (8510/3), PUCR (8014/3), SRCC (8490/3), UC (8020/3) and UCOGC (8035/3). Only cases with microscopically confirmed cancers were selected. Information was available on age, gender, tumor location, tumor stage, histological grade, surgical resection, chemotherapy, radiation and vital status. The SEER summary stage was used to achieve a uniform tumor classification over time. Cases with missing survival time were excluded. The SEER registry describes the histological differentiation of the tumor using a three grade system: G1, well differentiated; G2, moderately differentiated; G3, poorly differentiated or undifferentiated (anaplastic).

### Statistical analysis

Descriptive statistics were presented as percentages for categorical variables and median (interquartile range) for continuous variables. Chi-square or Kruskal–Wallis tests were used to compare patient, tumor and treatment characteristics across histological subtypes. The primary endpoint was overall survival. Kaplan–Meier curves were generated to estimate the probability of survival and compared between histological subgroups with the log-rank test. We estimated the hazard ratios (HRs) and 95% confidence intervals (CIs) for the associations between histological subtypes and survival adjusting for possible confounding variables by fitting a multivariable Cox proportional hazards regression model. Clinically relevant confounding variables were identified by univariable Cox proportional hazards regression analysis. Any variable with a p-value < 0.25 was selected as a candidate for the multivariable analysis. In the iterative process of variable selection, covariates were removed from the multivariable model if they were non-significant and not a confounder, as described by Hosmer-Lemeshow, resulting in a main effect model [12]. Missing values were imputed using multiple imputation with chained equations, as previously described [13]. The imputation method was predictive mean matching. The number of iterations for each chain was ten, as was the number of imputed data sets. STATA/MP 18.0 was used for all analyses.

## Results

Data were obtained from 228,157 patients with pancreatic cancer registered in the SEER database. Some 34,417 patients lacking microscopic confirmation of the tumor and 34,192 patients without a histological code of PDAC were excluded. The final study population comprised 159,548 patients with PDAC. The characteristics of the study population are shown in Tables 1 and 2. Median age was 68 years, and 48.2% were female. The median survival was 8 months. The 5-year survival rate was 5.3% for the entire population, 19.7% for operated patients and 1.5% for non-operated patients.

**Table 1:** Baseline characteristics of 159,548 patients with microscopically confirmed pancreatic ductal adenocarcinoma stratified by histological variant

Variables	cPDAC	CC	ASqC	SRCC	UC	UCOGC	HC	MCP	PUCR	*P*-value
N	153,028 (95.9%)	4,118 (2.6%)	1,346 (0.8%)	782 (0.5%)	150 (0.1%)	87 (0.1%)	23 (0.01%)	9 (0.006%)	5 (0.003%)	
Age, y (IQR)	68 (60–76)	68 (60–76)	68 (61–76)	68 (60–76)	67 (59–73)	66 (59–74)	65 (60–72)	65 (48–67)	61 (59–77)	0.223
Female	73,797 (48.2%)	2,068 (50.2%)	609 (45.2%)	315 (40.3%)	62 (41.3%)	44 (50.6%)	6 (26.1%)	6 (66.7%)	3 (60%)	< 0.001
Tumor location										< 0.001
Head	78,809 (51.5%)	1,856 (45.1%)	595 (44.2%)	365 (46.7%)	67 (44.7%)	39 (44.8%)	7 (30.4%)	4 (44.4%)	1 (20%)	
Body/tail	39,963 (26.1%)	1,143 (27.8%)	508 (37.7%)	196 (25.1%)	43 (28.7%)	32 (36.8%)	8 (34.8%)	2 (22.2%)	4 (80%)	
Other^a^	34,256 (22.4%)	1,119 (27.2%)	243 (18.1%)	221 (28.3%)	40 (26.7%)	16 (18.4%)	8 (34.8%)	3 (33.3%)	0 (0%)	
SEER summary stage										< 0.001
Localized	12,803 (8.4%)	442 (10.7%)	97 (7.2%)	28 (3.6%)	7 (4.7%)	20 (23.0%)	4 (17.4%)	0 (0%)	1 (20%)	
Regional	52,433 (34.3%)	1,223 (29.7%)	542 (40.3%)	216 (27.6%)	47 (31.3%)	36 (41.4%)	2 (8.7%)	8 (88.9%)	0 (0%)	
Distant	82,310 (53.8%)	2,337 (56.8%)	682 (50.7%)	509 (65.1%)	91 (60.7%)	31 (35.6%)	16 (69.6%)	1 (11.1%)	4 (80%)	
Unstaged	5,482 (3.6%)	116 (2.8%)	25 (1.9%)	29 (3.7%)	5 (3.3%)	0 (0%)	1 (4.3%)	0 (0%)	0 (0%)	
Grade										< 0.001
1	4,848 (3.2%)	415 (10.1%)	3 (0.2%)	1 (0.1%)	0 (0%)	0 (0%)	2 (8.7%)	0 (0%)	0 (0%)	
2	22,494 (14.7%)	651 (15.8%)	171 (12.7%)	32 (4.1%)	0 (0%)	0 (0%)	3 (13.0%)	0 (0%)	0 (0%)	
3	24,308 (15.9%)	424 (10.3%)	534 (39.7%)	330 (42.2%)	150 (100%)	87 (100%)	5 (21.7%)	8 (88.9%)	5 (100%)	
Unknown	101,378 (66.2%)	2628 (63.8%)	638 (47.4%)	419 (53.6%)	0 (0%)	0 (0%)	13 (56.5%)	1 (11.1%)	0 (0%)	
Surgical resection										< 0.001
Yes	30,356 (19.8%)	975 (23.7%)	496 (36.8%)	131 (16.8%)	43 (28.7%)	45 (51.7%)	6 (26.1%)	9 (100%)	1 (20%)	
No	121,059 (79.1%)	3,107 (75.4%)	838 (62.3%)	646 (82.6%)	107 (71.3%)	41 (47.1%)	17 (73.9%)	0 (0%)	4 (80%)	
Unknown	1613 (1.1%)	36 (0.9%)	12 (0.9%)	5 (0.6%)	0 (0%)	1 (1.1%)	0 (0%)	0 (0%)	0 (0%)	
Chemotherapy^b^	87,676 (57.3%)	2,304 (55.9%)	780 (57.9%)	385 (49.2%)	58 (38.7%)	58 (66.7%)	10 (43.5%)	5 (55.6%)	0 (0%)	< 0.001
Radiotherapy^c^	20,624 (13.5%)	517 (12.6%)	160 (11.9%)	73 (9.3%)	10 (6.7%)	19 (21.8%)	2 (8.7%)	2 (22.2%)	0 (0%)	< 0.001

^a^Other sites include C25.3, pancreatic duct; C25.7, other specified parts of pancreas; C25.8, overlapping lesion of pancreas; C25.9, pancreas, not otherwise specified. ^b^Chemotherapy data classified as “yes” or “no/unknown – no evidence of chemotherapy was found in the medical records examined”. ^c^Radiotherapy data classified as “yes” or “no/unknown – no evidence of radiation was found in the medical records examined”

**Table 2:** Survival estimates by histological variant

	cPDAC	CC	ASqC	SRCC	UC	UCOGC	HC	MCP	PUCR
Median survival (months)	8	9	7	5	5	12	5	41	2
5-year survival, by stage									
All stages	5.1%	10.6%	6.8%	3.4%	11.8%	28.4%	26.1%	33.3%	0%
Localized	13.8%	42.1%	15.2%	6.6%	22.2%	37.4%	100%	NA	0%
Regional	9.3%	16.0%	11.2%	7.9%	28.1%	40.0%	50%	37.5%	NA
Distant	1.2%	2.1%	2.5%	0.9%	3.4%	0%	0%	0%	0%
5-year survival, by surgical treatment									
Surgery	19.1%	36.0%	15.7%	19.0%	35.7%	45.7%	100%	33.3%	0%
No surgery	1.5%	2.4%	1.4%	0.2%	2.5%	4.8%	0%	NA	0%

### Conventional pancreatic ductal adenocarcinoma (cPDAC)

**Fig. 1:**
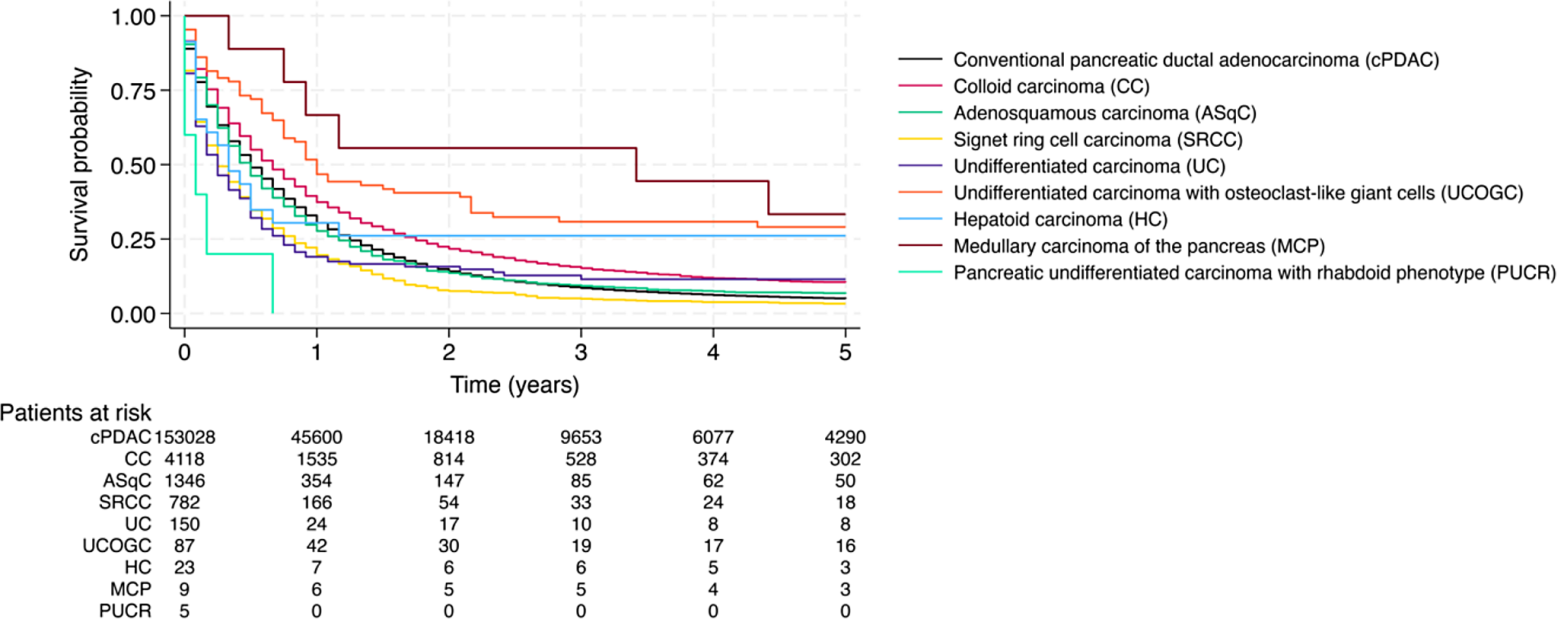
Kaplan–Meier analysis of patient survival stratified by histological variant (*p* < 0.001)

Conventional, or classical PDAC, consists of duct-like glandular structures within a desmoplastic stroma [14]. The median age at diagnosis was 68 years and 48.2% were female. The median survival was 8 months and the 5-year survival rate was 5.1% (Fig. 1).

### Colloid carcinoma (CC)

Colloid carcinomas, also known as non-cystic mucinous adenocarcinomas, are adenocarcinomas in which at least 80% of the neoplastic epithelium is scattered in extracellular mucin pools. CC is found in association with intestinal-type IPMNs [15]. The median age was 68 years and 50.2% were female. The median survival was 9 months and the 5-year survival rate was 10.6%. Compared to cPDAC, CC was associated with a better survival (multivariable HR: 0.78, 95% CI 0.76–0.81, *p* < 0.001); Tables 3 and 4.

**Table 3:** Univariable Cox regression analysis

Variables	HR	95% CI	*p*-value
Age	1.01	1.01–1.01	< 0.001
Female gender	0.99	0.98–0.99	0.033
Tumor location (head vs. others)	0.75	0.74–0.76	< 0.001
Histological grade (G3 vs. G1/G2)	1.49	1.46–1.51	< 0.001
Stage			
Localized	1		
Regional	1.04	1.02–1.06	0.001
Distant	2.35	2.30–2.40	< 0.001
Surgical resection	0.32	0.32–0.33	< 0.001
Chemotherapy	0.55	0.54–0.56	< 0.001
Radiotherapy	0.61	0.60–0.62	< 0.001
Histological subtype			
cPDAC	1		
CC	0.80	0.77–0.83	< 0.001
ASqC	1.03	0.97–1.10	0.316
SRCC	1.28	1.18–1.39	< 0.001
UC	1.07	0.88–1.31	0.490
UCOGC	0.52	0.40–0.67	< 0.001
HC	0.69	0.41–1.14	0.145
MCP	0.32	0.14–0.71	0.005
PUCR	2.78	0.90–8.62	0.077

CI, confidence interval; HR, hazard ratio

**Table 4:** Multivariable Cox regression analysis

Variables	HR	95% CI	*p*-value
Age	1.01	1.01–1.01	< 0.001
Female gender	0.95	0.94–0.96	< 0.001
Tumor location (head vs. others)	0.96	0.95–0.97	< 0.001
Grade (G3 vs. G1/G2)	1.30	1.28–1.32	< 0.001
Stage			
Localized	1		
Regional	1.35	1.32–1.38	< 0.001
Distant	2.26	2.21–2.31	< 0.001
Surgical resection	0.43	0.42–0.44	< 0.001
Chemotherapy	0.47	0.46–0.47	< 0.001
Radiotherapy	0.93	0.91–0.94	< 0.001
Histological subtype			
cPDAC	1		
CC	0.78	0.76–0.81	< 0.001
ASqC	1.16	1.09–1.22	< 0.001
SRCC	1.21	1.12–1.30	< 0.001
UC	0.99	0.83–1.18	0.912
UCOGC	0.68	0.53–0.87	0.002
HC	0.80	0.50–1.30	0.364
MCP	0.48	0.22–1.07	0.073
PUCR	1.54	0.64–3.69	0.337

CI, confidence interval; HR, hazard ratio

### Adenosquamous carcinoma (ASqC)

Adenosquamous carcinoma consists of at least 30% squamous differentiation with the rest of the tumor mass containing varying degrees of glandular adenocarcinoma [16]. The median age was 68 years and 45.2% were female. The median survival was 7 months and the 5-year survival rate was 6.8%. ASqC patients were resected more frequently (36.8%) in comparison to cPDAC (19.8%). The 5-year survival of patients with resected ASqC was 15.7%, which is lower than in resected cPDAC patients (19.1%). In the adjusted Cox model, ASqC was associated with a worse prognosis as compared to cPDAC (multivariable HR: 1.16, 95% CI 1.09–1.22, *p* < 0.001).

### Signet-ring cell carcinoma (SRCC)

Signet-ring cell carcinoma is comprised of at least 80% individual poorly cohesive cells, usually with intracellular mucin vacuoles and displaced nuclei [17]. The median age was 68 years and 40.3% were female. The median survival was 5 months and the 5-year survival rate was 3.4%. The prognosis of SRCC was inferior to cPDAC (multivariable HR: 1.21, 95% CI 1.12–1.30, *p* < 0.001).

### Undifferentiated carcinoma (UC)

Undifferentiated (anaplastic) carcinoma does not display any particular differentiation, such as glandular formation, mucin production or keratinization. Neoplastic cells are poorly cohesive and hypercellular and are found interspersed in a scant stroma [18]. The median age was 67 years and 41.3% were female. The median survival was 5 months and the 5-year survival was 11.8%. The prognosis of UC was not significantly different from cPDAC.

### Undifferentiated carcinoma with osteoclast-like giant cells (UCOGC)

These tumors are composed of pleomorphic, neoplastic mononuclear cells that are intermixed with non-neoplastic osteoclast-like multinucleated giant cells [19]. The median age was 66 years and 50.6% were female. The median survival was 12 months and the 5-year survival rate was 28.4%. UCOGC was associated with an improved survival compared to cPDAC (multivariable HR: 0.68, 95% CI 0.53–0.87, *p* = 0.002).

### Hepatoid carcinoma (HC)

Hepatoid carcinoma shows at least 50% hepatocellular differentiation. The tumor is composed of large polygonal cells with a rich eosinophilic granular cytoplasm and there may be an associated component of ductal adenocarcinoma [20]. The median age was 65 years and 26.1% were female. The median OS was 5 months and the 5-year survival rate was 26.1%. The prognosis of HC was not significantly different from cPDAC.

### Medullary carcinoma of the pancreas (MCP)

Medullary carcinoma of the pancreas is characterized by poor differentiation, a pushing border, syncytial cell growth and lymphocytic reaction [21]. The median age was 65 years and 66.7% were female. MCP had the highest survival rate among the tumor subtypes with a median survival of 41 months and a 5-year survival rate of 33.3%. MCP showed a trend towards being a predictor of better prognosis as compared to cPDAC (multivariable HR 0.48, 95% CI: 0.22–1.07, *p* = 0.073).

### Pancreatic undifferentiated rhabdoid carcinomas (PUCR)

Pancreatic undifferentiated rhabdoid carcinomas, in the WHO classification termed “large cell carcinomas with rhabdoid phenotype”, display abundant pleomorphic neoplastic giant cells with abundant eosinophilic cytoplasm and rhabdoid inclusions [22]. The median age was 61 years and 60% were female. PUCR displayed the worst prognosis among the tumor subtypes, with a median survival of 2 months and a 5-year survival rate of 0%. There were only 5 patients with PUCR in the cohort.

## Discussion

PDAC can be classified into several morphological variants. However, whether these histological variants have distinct biological behavior and alter prognosis have hitherto been unclear. Previous studies have investigated individual subtypes [23–33]. For MCP, HC and PUCR only case reports exist [20, 34, 35]. The current study is the first to include all known histological PDAC subtypes and compare the clinical features and prognostic impact of each morphological entity to cPDAC.

We found that cPDAC represented most (95.6%) PDACs and other histological variants were uncommon (4.4%). The vast majority of tumors were detected at late stages, highlighting the importance of early detection strategies to improve outcomes. PUCR had the worst prognosis, while MCP had the best prognosis. CC, ASqC, SRCC and UCOGC were identified as independent predictors of overall survival. For certain very rare subtypes, the small number of cases may have resulted in the non-significant HRs in the multivariable analysis.

CC was more indolent than cPDAC and associated with a better outcome, which confirms previous findings [23–25]. CC typically arises in the setting of IPMN [36]. The SEER database does not provide information on the presence of concomitant IPMN in CC or cPDAC. However, no difference in survival outcomes between cPDAC and cPDAC with concomitant IPMN after multivariable adjustment has been found [37].

ASqC had a similar survival compared to cPDAC in the unadjusted survival analysis, but the results indicated that ASqC was a predictor of poor prognosis after multivariable adjustment. We attribute this discrepancy to the high resection rate of ASqC (36.8%). This is line with prior work, suggesting that ASqC tumors are more aggressive biologically, and where subgroup analyses of resected patients have shown a significantly worse overall survival for ASqC patients as compared to cPDAC patients [26–28].

SRCC was associated with more advanced histopathological features and a worse prognosis when compared to cPDAC. It has previously been demonstrated that SRCC is a highly aggressive neoplasm with poor prognosis and the lungs as a preferred metastatic site [29–32]. The signet-ring cell component (> 80%) appears to be an adverse prognostic marker. Genomic analyses have revealed similarities to cPDAC regarding classical genetic drivers, such as KRAS, TP53, SMAD4, and CDKN2A, but also major differences, such as EGFR alterations, RET::CCDC6 fusion gene, and microsatellite instability [30].

The WHO classifies undifferentiated carcinoma (UC) of the pancreas into tumors that harbor osteoclast-like giant cells (UCOGC) and those that do not (UC/anaplastic). The clinical behavior of UCOGC appears to be unpredictable, but many behave unexpectedly well and the prognosis is better than UC/anaplastic [33]. Tumors defined as UC/anaplastic may be further subclassified into giant cell carcinoma, pleomorphic large cell carcinoma, and sarcomatoid carcinoma [9]. These additional subtypes of UC are associated with varying prognosis, further highlighting the importance of histological subtyping [33].

HC, MCP and PUCR are all extremely rare neoplasms of the pancreas, representing less than 0.01% of all PDACs in our study. The natural history and prognosis of these rare subtypes of PDAC may not be accurately predicted due the low case numbers. However, we noted that HC had a better 5-year survival rate than cPDAC after surgical resection, but for the unresectable group the prognosis was similar to that for unresectable cPDAC, which is consistent with a previous compilation of reported cases in the literature [20]. Our data also indicated that PUCR is a rapidly fatal neoplasm, while MCP has a more indolent course, confirming data from case reports [34, 35].

Variant histology is prone to being underrecognized or misclassified. This is exemplified by a previous report where PUCR was initially suspected to be a solid pseudopapillary neoplasm [38]. Furthermore, there exists significant intratumoral heterogeneity and if representative regions are not sampled, a patient may be incorrectly classified [20]. For example, in ASqC, the arbitrary limit of 30% squamous component could lead to misdiagnosis of cPDAC on simple tissue biopsy [39].

With the increasing knowledge of the molecular landscape of PDAC [40], histological appearance may not be enough to subclassify tumors. Purely molecular [41] or morphological models [42] exist to predict treatment response, but lack whole-tumor characterization. Future diagnostic algorithms may utilize molecular profiling in addition to morphological appearance in order to improve diagnostic classification. An example of such an diagnostic algorithm is “Pancpaint”, which associates the classical and basal-like molecular subtypes with a specific morphology and can be applied to whole tissue-slides [43].

There are several limitations to this study. This was a retrospective study of a multi-institutional registry. We included only microscopically confirmed cases, which improved reliability of the data, but may have contributed to the high resection rate observed in the patient material. The SEER registry may also underestimate treatments, such as chemotherapy and radiation [44]. There may also be inherent flaws with the pathologic identification and histological coding of the rare tumor subtypes.

## Conclusion

In 159,548 patients with PDAC, histological subtyping provided important prognostic information. Patients with ASqC and SRCC had inferior survival, while patients with CC and UCOGC had superior survival compared to cPDAC after adjusting for confounding factors. Considering these results, variant histology should become an important part of risk stratification for patients with PDAC. In the future, molecular characterization of variant histology could potentially help predict responders to treatment and underly development of new therapies.

## Data Availability

The data that support the findings of this study are available from the corresponding author upon reasonable request.
